# Roles and Therapeutic Implications of Endoplasmic Reticulum Stress and Oxidative Stress in Cardiovascular Diseases

**DOI:** 10.3390/antiox10081167

**Published:** 2021-07-22

**Authors:** Yan Zhou, Dharmani Devi Murugan, Haroon Khan, Yu Huang, Wai San Cheang

**Affiliations:** 1 State Key Laboratory of Quality Research in Chinese Medicine, Institute of Chinese Medical Sciences, University of Macau, Macau 999078, China; yc07517@um.edu.mo; 2 Department of Pharmacology, Faculty of Medicine, University of Malaya, Kuala Lumpur 50603, Malaysia; dharmani79@um.edu.my; 3 Department of Pharmacy, Abdul Wali Khan University, Mardan 23200, Pakistan; haroonkhn@awkum.edu.pk; 4 School of Biomedical Sciences, Chinese University of Hong Kong, Hong Kong 999077, China; yu-huang@cuhk.edu.hk

**Keywords:** endoplasmic reticulum stress, oxidative stress, cardiovascular diseases

## Abstract

In different pathological states that cause endoplasmic reticulum (ER) calcium depletion, altered glycosylation, nutrient deprivation, oxidative stress, DNA damage or energy perturbation/fluctuations, the protein folding process is disrupted and the ER becomes stressed. Studies in the past decade have demonstrated that ER stress is closely associated with pathogenesis of obesity, insulin resistance and type 2 diabetes. Excess nutrients and inflammatory cytokines associated with metabolic diseases can trigger or worsen ER stress. ER stress plays a critical role in the induction of endothelial dysfunction and atherosclerosis. Signaling pathways including AMP-activated protein kinase and peroxisome proliferator-activated receptor have been identified to regulate ER stress, whilst ER stress contributes to the imbalanced production between nitric oxide (NO) and reactive oxygen species (ROS) causing oxidative stress. Several drugs or herbs have been proved to protect against cardiovascular diseases (CVD) through inhibition of ER stress and oxidative stress. The present article reviews the involvement of ER stress and oxidative stress in cardiovascular dysfunction and the potential therapeutic implications.

## 1. Introduction

Endoplasmic reticulum (ER) is a crucial organelle in which protein synthesis, maturation, folding and trafficking take place. Only properly folded proteins can be destined to cellular organelles or cell surface; nevertheless, misfolded or unfolded proteins are retained in the ER to be degraded eventually [1]. Disruption of the aforementioned processes results in the accumulation of newly synthesized unfolded proteins in the ER and this condition is referred to as ER stress [2]. ER stress occurs in different pathological conditions including ischemia, hypoxia, altered glycosylation, nutrient deprivation, oxidative stress and Ca^2+^ depletion of ER stores, and consequently activates ER membrane-associated proteins and complex downstream signaling pathways to regulate targeted gene expression [3]. In addition, ER chaperone proteins promote the folding of other proteins, prevent protein aggregation under ER stress and maintain the normal ER function [4]. Some chaperone proteins have a positive effect on cardiovascular disease (CVD). For example, the 70 kDa heat shock protein (Hsp70) chaperones showed cardioprotective effects in ischemia/reperfusion animal models [5], and dysregulation of glucose regulatory protein 78 (GRP78) chaperones is associated with atherosclerosis [6]. Moreover, unfolded or misfolded proteins in the ER can be degraded and quickly eliminated by the ubiquitin-proteasome system (UPS), which is called ER-Associated Degradation (ERAD). UPS and ER jointly maintain cell homeostasis and participate in the major pathways of signal transduction, cell cycle regulation and protein degradation [7]. Proteasome inhibitors have been shown to reduce inflammation and protect vascular function in models of atherosclerosis [8].

Oxidative stress is mainly caused by imbalanced production of reactive oxygen species (ROS) and antioxidants [9,10]. ROS are highly reactive chemical substances containing oxygen, controlled by the activities of specific antioxidant enzymes, such as catalase, glutathione peroxidase and superoxide dismutase [11]. The level of ROS is strictly controlled, where it is harmful at high concentrations but beneficial at low concentrations [12]. Under normal physiological conditions, the low concentrations of ROS play an important role in cell homeostasis by regulating cell signal transduction and physiological activity [13]. However, when the production of ROS exceeds the control of the antioxidant defense mechanisms, oxidative stress will occur, hindering protein folding, damaging the synthesis and accumulating toxic products, which affect the normal function of cells and lead to cell death, and ultimately may lead to diseases in the cardiovascular system [9,14,15,16].

CVD is a common multifactorial disease [17,18]. CVD including hypertension, pulmonary hypertension, myocardial infarction and stroke is mainly caused by atherosclerosis; obesity and diabetes are major risk factors [19,20]. Studies have demonstrated that ER stress and oxidative stress play roles in the pathogenesis of these cardio-metabolic diseases. The present article reviews the involvement of ER stress and oxidative stress in cardiovascular dysfunction and the potential therapeutic implications.

## 2. Regulation of ER Stress and Integration with Other Signaling Networks

In response to ER stress, the three branches of the unfolded protein response (UPR) are activated, initiated by three ER membrane-associated proteins—PKR-like ER kinase (PERK), inositol requiring enzyme 1 (IRE1) and activating transcription factor 6 (ATF6)—and engaged complex downstream signaling pathways [2,19]. In the first branch, phosphorylated PERK phosphorylates eukaryotic initiation factor 2 on the alpha subunit (eIF2α) at Ser51 that attenuates mRNA translation. In addition, the PERK-dependent phosphorylation results in the activation of transcriptional factors: ATF4, nuclear erythroid 2-related factor 2 (Nrf2) and nuclear factor kappa-light-chain-enhancer of activated B cells (NF-κB) [21,22]. In the second branch, autophosphorylated IRE1 recruits tumor necrosis factor (TNF) receptor-associated factor 2 (TRAF2) to activate Jun N-terminal kinase (JNK) and inhibitory kappa B kinase (IKK), and the downstream transcription factors activator protein 1 (AP1) and NF-κB [23]. Activated IRE1 also removes a 26-base intron from the mRNA of X-box binding protein 1 (XBP1). This spliced *XBP1* mRNA then translates into active transcriptional factor XBP1 [24]. In the third branch, ATF6 is released and translocated to the Golgi apparatus, where it is cleaved to yield an active cytosolic ATF6 fragment acting as transcription factor [25]. The primary role of UPR is to protect the cell from ER stress by inducing the transcription of genes encoding ER chaperones and enzymes that promote protein folding, maturation, trafficking and ER-associated protein degradation so as to remove the accumulated misfolded proteins in the ER [26,27]. However, if the cell fails to clear up the protein-folding defect and restore homeostasis in the ER, the UPR will trigger apoptosis instead to eliminate the stressed cells [28].

ER stress-induced apoptosis is mediated by C/EBP homologous protein (CHOP), a transcriptional factor downstream of the PERK/eIF2α/ATF4 and the ATF6 pathways [29]. Furthermore, IRE1-TRAF2 complex interacts with apoptosis signal-regulating kinase 1 (ASK1) which subsequently phosphorylates c-Jun N-terminal kinase (JNK) [30]. Both CHOP and JNK influence the apoptotic machinery by regulating the balance between pro-apoptotic and anti-apoptotic proteins from the B-cell lymphoma-2 (Bcl-2) family [31]. CHOP blocks the expression of anti-apoptotic Bcl-2 while it upregulates pro-apoptotic Bcl-2 homology domain 3 (BH3)-only members of the Bcl-2 family [32]. Activation of JNK phosphorylates Bcl-2, suppressing the anti-apoptotic activity of Bcl-2. JNK also phosphorylates BH3-only proteins to enhance their pro-apoptotic potential [33].

Increasing evidence shows that ER stress/UPR pathways link with other signaling networks. Pivotal inflammatory mediator, NF-κB, is activated by the IKK pathway during ER stress, inducing production of proinflammatory cytokines such as interleukin (IL)-6, IL-8, monocyte chemotactic protein 1 (MCP-1) and TNF-α [34]. ER stress also triggers activation of JNK/AP1 and induces expression of inflammatory genes. Furthermore, mitochondria forming close complex with ER through ER-associated mitochondria membranes (MAMs) are important sources of ROS [35], and thus mitochondria-derived ROS promotes ER stress [36]. Importantly, ROS is produced through the mutual redox reaction in the three organelles of mitochondria, peroxisomes and ER, known as the “Redox triangle” [37]. The accumulated ROS affects Ca^2+^ transfer and the protein folding function of ER stress. Mitochondrial dysfunction is aggravated by ER stress, causing massive loss of mitochondrial membrane potential, oxidative phosphorylation and other conditions [38,39]. During ER stress, a large amount of activated JNK binds to the MAM linking protein Sab and releases ROS with mitochondria [40]. The carrier protein uncoupling protein-2 (UCP-2) on mitochondria also maintains normal mitochondrial function, thus maintaining NO homeostasis and protecting the endothelial dysfunction in diabetes and obesity [41]. Ca^2+^ signaling molecules can tightly connect mitochondria and ER through locally inhibiting mitochondrial movement and promoting their distribution around the ER, enhancing the production of Ca^2+^ and ATP, thereby activating intrinsic apoptosis [42]. Furthermore, ROS enhances the formation of inflammasomes on MAMs which then induce the generation of IL-1β and IL-18 and the downstream inflammatory response [43]. It is also important to note that TNFα-induced ROS accumulation causes ER stress [44].

**Figure 1:**
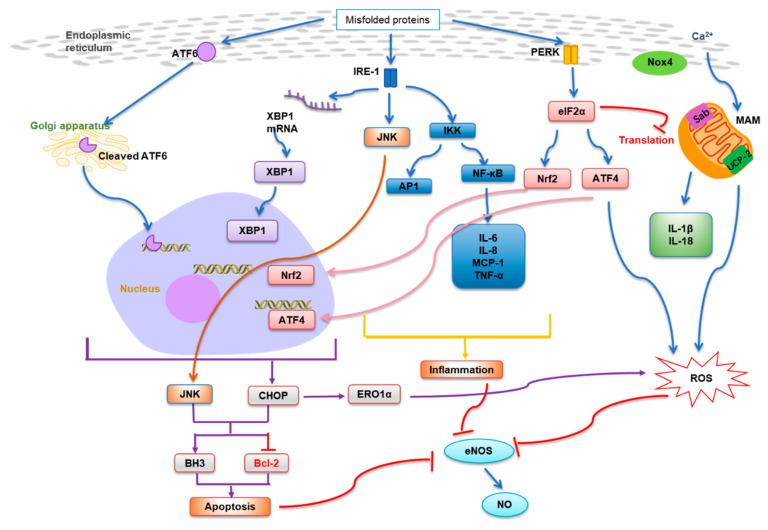
Schematic diagram showing regulation of endoplasmic reticulum (ER) stress and integration with other signaling networks. ER stress leads to inflammation, apoptosis, oxidative stress and reduced nitric oxide (NO) bioavailability, affecting vascular homeostasis.

On the other hand, ER stress is the main source of ROS and plays an important role in oxidative stress [45]. Stressed ER produces more ROS as well as fewer antioxidants due to the inhibition of protein translation via PERK/eIF2α/ATF4 pathway [46]. This stress condition can be partially mitigated by the ATF4-induced antioxidant pathway involving glutathione and by activation of the Nrf2-mediated antioxidant pathway [47]. In response to oxidative stress, PERK activates the antioxidant transcription factor Nrf2 to dissociate from its inhibitor Keap-1 and transfer into the nucleus, thereby increasing intracellular glutathione levels and activating the transcription of cell defense genes [48]. ROS generation is elevated during ER stress via NADPH oxidases, mainly Nox2 and Nox4 [49]. Nox4 is mainly activated through PERK/eIF2α/ATF4 and IRE1-dependent XBP1 splicing pathway. In the late stage of ER stress, the increase in the expression of ERO1α induced by the transcription factor CHOP will increase the level of ROS [50]. Notably, endothelial nitric oxide synthase (eNOS), an important enzyme modulating vascular homeostasis, produces NO to protect blood vessels, and its activity is diminished by ER stress-induced apoptosis and inflammation [51], resulting in a reduction in NO bioavailability and thereby vascular dysfunction [48,52,53]. These results reveal the complex linkages among ER stress/UPR, oxidative stress and inflammation, modulating vascular homeostasis (Figure 1).

## 3. ER Stress Linking to Cardiovascular Complications in Diabetes and Obesity

Excess nutrients and inflammatory cytokines associated with metabolic diseases can trigger or worsen ER stress [54]. High glucose [55], free fatty acids (FFAs) [56], oxidized and glycated low-density lipoproteins (LDLs) [57] have been reported to induce ER stress [58]. ER stress markers PERK, IRE1α, ATF6α and JNK are activated in obese (*ob*/*ob*) mice and in mice fed a high-fat diet [59,60]. Chemical chaperones such as 4-phenyl butyric acid (PBA) and taurine-conjugated ursodeoxycholic acid (TUDCA) are known to alleviate ER stress. Treatment of *ob*/*ob* and diabetic (*db*/*db*) mice with PBA and TUDCA normalizes hyperglycemia, increases insulin sensitivity and reduces fatty liver disease [61]. The latest research shows that nicorandil, an approved drug for treating angina with vasodilatory property, can inhibit the PERK pathway induced by ROS-ER stress to ameliorate insulin resistance caused by high glucose [62].

Studies have suggested that 5′ adenosine monophosphate-activated protein kinase (AMPK) is a physiological suppressor of ER stress [63,64]. Apart from regulating systemic energy balance and metabolism [65], AMPK activation protects endothelial function which is attributed to inhibiting proliferation of vascular smooth muscle cells (VSMCs) and increasing nitric oxide (NO) production in endothelial cells [66,67]. Pharmacological or genetic activation of AMPK was shown to mitigate ER stress in endothelial cells and enhance endothelium-dependent relaxation in mouse aortas [57,68]. In addition, oxidative stress may be linked with ER stress. Inhibition of ROS production by blocking Rac1 or NADPH oxidase normalizes the elevated expression of ER stress markers and improves myocardial function in type 1 diabetic mice; a similar phenomenon is observed in diabetic retinopathy, diabetic nephropathy and atherosclerosis [69,70,71,72]. These findings suggest that ER stress and oxidative stress are potential mechanisms involved in vascular complications associated with obesity and diabetes.

Notably, AMPK subunits α1 and α2 have been reported to bind with PPARδ to form a transcriptional complex and subsequently induce the transcription of several PPAR target genes such as uncoupling protein 3 and lipoprotein lipase [73]. Peroxisome proliferator activated receptors delta (PPARδ) is ubiquitously expressed, for example, in adipocytes, VSMCs and endothelial cells [74,75,76]. PPARδ activation stimulates fatty acid β-oxidation in adipocytes and skeletal muscle, depletes lipid accumulation and attenuates obesity [77,78]. PPARδ also regulates glucose homeostasis [79,80], as well as protects against atherosclerosis [81] and endothelial dysfunction [82,83,84].

A widely used anti-diabetic drug metformin improves insulin sensitivity and glucose homeostasis, and at the same time reduces endothelial dysfunction [85,86] and thereby cardiovascular risks in diabetic patients [87]. Metformin is well known to activate AMPK in different tissues in humans and rodents [88,89,90]. The effects of metformin on endothelial function are largely mediated through AMPK and PPARδ with a subsequent alleviation of ER stress and oxidative stress as well as increased eNOS activity and NO production, highlighting the central role of PPARδ downstream of AMPK activation to combat against diabetes- and obesity-related vasculopathy, inflammation and hypertension [91,92,93]. Likewise, exercise is known to activate AMPK and is found to ameliorate ER stress and endothelial dysfunction in diabetes through PPARδ [94]. These findings imply a close linkage between ER stress and vascular dysfunction in diabetes modulated by AMPK and PPARδ.

Evidence shows that ER stress contributes to endothelial cell inflammatory responses and apoptosis in diabetic retinopathy [95,96]. In streptozotocin (STZ)-induced type 1 diabetic mice, retinal inflammation and vascular leakage are attributed to ATF4 activation of STAT3 [97,98]. In addition, elevated epidermal growth factor receptor tyrosine kinase (EGFR-TK) phosphorylation activates PERK/eIF2α/ATF4 branch and contributes to microvascular dysfunction as well as cardiac fibrosis in the STZ model [99,100]. Treating STZ mice with EGFR-TK inhibitor (AG1478) and ER stress alleviator (TUDCA) augments endothelium-dependent relaxation and reverse the augmented Nox2 and Nox4 in mesenteric resistance arteries [101]. Importantly, TUDCA treatment exerts protective effects on hind-limb ischemia coupled with reduced body weight, blood glucose and insulin level in type 2 diabetic *db*/*db* mice [102]. These suggest that ER stress plays a role in ischemia-induced neovascularization associated with diabetes and is involved in regulating obesity and insulin resistance.

Mangiferin, a widely used Chinese herb for treating diabetes mellitus, has been shown to inhibit ER stress and the associated oxidative stress, TXNIP expression, NLRP3 inflammasome activation, production of IL-1β and IL-6 whilst increasing NO release in endothelial cells exposed to high glucose [103,104]. In addition, physical exercise has been shown to have vasoprotective effect, activating PPARδ to improve vascular endothelial function in diabetic and obese mouse models [94].

## 4. ER Stress in Atherosclerosis

In line with the in vitro results that high glucose induces ER stress markers and accompanied endothelial dysfunction [55,105], FFAs [56], oxidized and glycated LDLs [57], ER stress has been reported to be activated at athero-susceptible arterial regions [106] in obese (*ob*/*ob*) mice and in mice fed a high-fat diet [59]. On the other hand, reduction in AMPKα2 expression increases the ER stress and atherosclerosis [107].

In view of the high prevalence of obesity clustering with type 2 diabetes and CVD, the detailed mechanisms of its pathogenesis and effective therapeutic approaches are important to explore nowadays. In contrast to proinflammatory effects of oxidized LDLs (oxLDLs), high-density lipoproteins (HDLs) have anti-inflammatory and antioxidant effects and they can prevent ER stress and autophagy induced by oxLDL [108,109,110].

IRE1 inhibitors STF-083010 and 4μ8C suppress lipid-triggered mitochondrial ROS release, NLRP3 inflammasome activation, production of IL-1 and IL-18 and thus retarding the progression of atherosclerosis in ApoE^−/−^ mice [111].

Some natural compounds have also been shown to inhibit ER stress, implying a protective role in atherosclerosis. Kaempferol acts on caspase-3 and caspase-7 and downregulates the expression of GRP78 and CHOP to prevent cell death [112]. Atrovastatin inhibits ER stress through AMPK activation in human endothelial cells and in atherosclerotic mice [113]. In addition to natural compounds, exercise has been shown to attenuate ER stress, thereby ameliorating atherosclerotic vascular dysfunction by reversing the increase in the expression of CHOP in atherosclerotic mice and increasing the expression of downstream signaling pathways, including eNOS, UCP-2 and caspase-1 [114].

## 5. Hypertension

ER stress has been strongly implicated in the pathogenesis of hypertension, involving diverse cardioregulatory systems including brain, pulmonary and systemic vasculature and heart [45,115]. ER stress in brain has been implicated in the angiotensin II (Ang II)-induced hypertension which can be reversed by treatment with TUDCA [116]. CNS selective administration of TUDCA globally reduces brain ER stress and rescues obesity-induced hypertension [117]. Infusion of Ang II in mice induced the UPR proteins BiP and CHOP in aortas, mesenteric arteries and myocardium [118]. The same group demonstrated that both TUDCA and 4-PBA normalize blood pressure, reduce cardiomyocyte UPR activity and cardiac fibrosis and restore macrovascular endothelial function via inhibition of transforming growth factor-beta 1 (TGF-β1), confirming a key pathogenic role for ER stress in hypertension [118]. Similar results were obtained in hypertensive rats whereby ER stress inhibition by 4-PBA and TUDCA normalized blood pressure by suppressing Ca^2+^-dependent cytosolic phospholipase A2 (cPLA2)/cyclooxygenase (COX) pathway [119]. Additional work has also shown an improved vascular function with decreased Ang II-induced vascular ER stress [120,121,122,123]. These studies showed that inhibition of ER stress is partially attributed to the decrease in Ang II-mediated markers of oxidative stress such as nitrotyrosine, NOX 2 and NOX4. It is likely that during hypertensive disease, vascular oxidative and ER stress are bi-directionally related. Furthermore, Takayanagi et al. demonstrated that inhibition of metalloproteinase domain 17 (ADAM17) activation contributes to the diminished Ang II-induced ER stress [124].

## 6. Pulmonary Arterial Hypertension

Pulmonary arterial hypertension (PAH) is related to excessive pulmonary vasoconstriction and abnormal vascular remodeling. Previous studies showed that ER stress causes vascular pathological changes and thus participates in the occurrence and development of PAH [125]. Research targeting ER stress in PAH has focused on pulmonary artery smooth muscle cells (PASMC), as exaggerated proliferation and resistance to apoptosis of PASMCs is a key component of vascular remodeling [126]. ER stress has been found to be activated in the vasculature of mice with hypoxia-induced PAH [127], and administration of 4-PBA significantly reduces pulmonary hypertension, arterial remodeling and right ventricular hypertrophy [128,129,130]. Besides the known chemical chaperones, hydrogen sulfide [115], intermedin [131] and 4u8c, an inhibitor of the IRE1α/XBP1 pathway [132], have been shown to restrain hypoxia-induced cell proliferation and migration and reverse hypoxia-induced apoptosis arrest, which correlated with the suppressed expression of UPR markers. In 2011, Sutendra et al. found that the lack of Nogo-B in PASMCs from Nogo-A/B-deficient mice prevents hypoxia-induced changes in vitro and in vivo, resulting in complete resistance to induction of PAH [133]. Nogo is a member of the reticulon family of proteins that regulates the tubular structure of the ER and is implicated in vascular remodeling and PAH. The elevated levels of Nogo-B during ER stress lead to restructuring and disruption of the mitochondria-ER unit, which eventually suppress apoptosis; thus, targeting Nogo-B can be a potential selective therapeutic strategy against PAH [133,134]. Of note, mice with conditional deletion of GATA-6, a member of the GATA family of zinc-finger transcription factors, in endothelial cells display an elevation of ER stress markers and worsening of hypoxia-induced PAH [135,136]. This result reveals that endothelial cells play a critical role in triggering ER stress in hypoxic mice. In peripheral blood mononuclear cells isolated from patients with limited cutaneous systemic sclerosis and PAH, ER stress markers are upregulated and in positive correlation with IL-6 level and severity of pulmonary artery pressure [137].

## 7. Hyperhomocysteinemia

Homocysteine (Hcy) is well known to induce ER stress [138]. High plasma level of Hcy represents an independent risk factor for CVD [139,140]. Hcy induces cell death of vascular endothelial cells by activation of JNK and ATF3 through IRE1/TRAF2 pathway [141] and by eIF2α induction of the T-cell death associated gene 51 (TDAG51) [142] which contributes to the development of atherosclerosis. Hcy-induced ER stress is also shown to impair NO production and calcium-activated potassium channels (IK_Ca_ and SK_Ca_) [143] and increase NOX-generated ROS [50] to reduce vasorelaxation. In addition, Hcy was shown to target soluble epoxide hydrolase (sEH), a major enzyme that hydrolyzes epoxyeicosatrienoic acids and attenuates their cardiovascular protective effects [144]. Treatment of cultured human endothelial cells with Hcy upregulates sEH levels, which is associated with the up-regulation of adhesion molecules and activation of ATF6 [145,146]. Besides Hcy, homocysteine thiolactone was also shown to upregulate GRP78 expression and induce ER stress, leading to a downstream enhancement of oxidative stress and inflammation, which finally cause vascular endothelial dysfunction [147]. Overall, Hcy-induced ER stress and oxidative stress are reciprocally associated.

Hyperhomocysteinemia (HHcy) is associated with hypertension. Alleviation of ER stress by black tea consumption was found to ameliorate vascular dysfunction and normalize plasma Hcy level and blood pressure in hypertensive rats [120]. Enalapril, an antihypertensive agent, was shown to protect endothelial cells and improve hypertension through inhibiting ER stress, as well [148]. Piceatannol, a resveratrol analogue, protected endothelial cells against Hcy-induced apoptosis, oxidative stress and ER stress via Nrf2-dependent expression of heme oxygenase 1 [149]. In hyperhomocysteinemic mice, atorvastatin improved atherosclerotic plaque stability by inhibiting ER stress [150] and this protective effect may involve activation of AMPK [151]. Moreover, salidroside protects against HHcy-induced endothelial dysfunction by down-regulating Bip and CHOP expression and decreasing PERK and IRE1α phosphorylation [152]. Alpha-lipoic acid ameliorates Hcy-induced ER stress and oxidative stress, thus reducing apoptosis and inflammation in human aortic endothelial cells [153]. The glucagon-like peptide 1 (GLP-1) analogue exendin-4 activates AMPK, inhibits ER stress and superoxide anion production, thereby ameliorating HHcy-induced endothelial dysfunction [154]. These findings suggest that targeting Hcy-induced ER stress is a potential therapeutic strategy for treating CVD.

## 8. Myocardial Infarction

Left ventricular remodeling after myocardial infarction (MI) is a key factor in heart failure [155]. MI activates JNK through the IRE1/TRAF2 pathway, showing high levels of JNK phosphorylation [156]. ASK1 plays an important role in ER stress-induced apoptosis [157]. Deletion of ASK1 attenuates left ventricular remodeling, implying that ER stress contributes to myocyte loss during MI [158]. MI activates mitogen-activated protein kinase (MAPK) through induction of ER stress, and NFκB pathway in hypoxic cardiac cells or cardiac infarct tissues [159]. In the MI mouse model induced by left anterior descending (LAD) ligation, the expression of inflammatory cytokines in the heart increased, such as TNFα, IL-1, IL-6 and MCP-1 [160]. In addition, the expression of Bax and cleaved caspase 3 also elevated, while the expression of Bcl-2 diminished in the treated mice. MI can induce oxidative stress, leading to increased ROS accumulation in hypoxic cardiac cells and the elevated activity of NADPH oxidase [161].

Zinc finger protein ZBTB20 has an anti-apoptotic effect and can relieve heart remodeling after MI. ZBTB20 protects the heart by reducing the expression of TNFα, inhibiting ASK1 and JNK signal transduction, and also suppressing the activity of NADPH oxidase [161]. Valsartan improves myocardial remodeling by inhibiting ASK1-dependent signaling pathway [162]. *Panax notoginseng* flower (PN-F) is widely used to treat CVD by increasing expression of genes including hypoxia inducible factor 1 (HIF-1), vascular endothelial growth factor-α (VEGFA) and kinase insert domain receptor (KDR), and upregulating the expression of Bcl-2 and Bax to inhibit cell apoptosis and promote angiogenesis in the infarct area [163]. Furthermore, exercise can be used as an adjuvant treatment for heart failure, thereby improving CVD by increasing the activity of the anti-aging molecule Sirt 1 [164]. All this evidence supports the interaction among ER stress, oxidative stress and inflammation contributing to MI and the potential therapeutic implications such as using valsartan, natural plants (PN-F) and exercise.

## 9. Stroke

In the case of stroke, ER stress-associated apoptosis is induced and leads to neuronal cell death, which can be prevented in CHOP-deficient mice [165]. Stroke induces IRE1 and PERK to enhance the expression of GRP78 and CHOP through ER stress to promote cell apoptosis [166]. Ischemic stroke is the most common type of stroke and the disease with the most serious sequelae [167]. A recent study shows that Hes1 knockdown induces cell apoptosis through ER stress, which can increase cerebral infarction and aggravate ischemic stroke. Hes1-induced ER stress can exacerbate the disease by activating the PERK/CHOP signaling pathway [168]. Taurine has been shown to inhibit the pathways of ATF6 and IRE1 and limits ROS-induced ER stress in rat stroke model [169]. Apelin-36 protects neurons from apoptosis and activates caspase-3 by inhibiting the activation of ER stress and UPR and the increase in CHOP/GRP78, thereby reducing infarction and cell apoptosis caused by ischemic stroke [170]. The basic fibroblast growth factor (bFGF) can improve ischemic oxidative damage by inhibiting ER stress response proteins including CHOP and ATF6. In addition, bFGF achieves neuroprotective effects by activating PI3K/Akt and ERK1/2 pathways [171].

## 10. ER Stress as Drug Target to Combat against CVD

Given the evidence that ER stress is widely associated with CVD, pharmacological modulation to manipulate ER stress/UPR signaling becomes a growing consensus to treat diverse vascular disorders [172]. Approaches to prolong the adaptive phases of the UPR to enhance cell survival and recovery or to inhibit ER stress-associated apoptosis may be beneficial in combating against a range of human diseases [173]. For instance, salubrinal was found to inhibit eIF2α dephosphorylation and thus favors cell survival under ER stress [174]. FDA-approved 26S proteasome inhibitors, bortezomib and carfilzomib induce ER stress and cell death in multiple myeloma cells and are used for the treatment of multiple myeloma [175]. On the other hand, 4-phenylbutryic acid (PBA) and taurine-conjugated ursodeoxycholic acid (TUDCA) are shown to attenuate ER stress and disease symptoms in animal models. PBA and TUDCA are FDA-approved chemical chaperones for clinical use in urea cycle disorders and in cystic fibrosis, respectively. Oral administration of PBA and TUDCA ameliorates glucose homeostasis and defects in pancreatic β cells in diabetic muse models [61,176]. More importantly, both chemical chaperones ameliorate insulin resistance in human obese subjects [177,178]. Another chemical chaperone SRT1720 reduces ER stress, apoptosis and inflammation induced by glucosamine in HUVECs through the regulation of raptor acetylation [179]. Importantly, the drugs approved to treat hypertension, obesity and diabetes including enalapril, valsartan, atorvastatin, metformin and exendin-4, as well as herbal medicine or natural plants such as mangiferin, black tea and *Panax notoginseng* flower, have been demonstrated to possess vasoprotective effects through inhibiting ER stress and oxidative stress. Besides natural compounds, physical activity and exercise-based cardiac rehabilitation are effective to suppress oxidative stress and thus improve endothelial function. Exercise improves vascular function by regulating the balance between NO and ROS. Some key mediators of motor regulation may provide a potential effective target for the treatment of CVD. These studies suggest the therapeutic potential of targeting ER stress to treat human disease. In regard to the cross-wired networking of the UPR/ER stress pathways with other signaling networks, multiple nodes should be targeted simultaneously to achieve desired benefits.

## 11. Conclusions and Perspectives

Taken all together, increasing evidence indicates the crucial role of endoplasmic reticulum (ER) stress in vascular dysfunction. It is likely that occurrence of ER stress contributes to the reduced nitric oxide (NO) bioavailability and thereby vascular dysfunction through oxidative stress- and inflammation-dependent mechanisms. In addition, many drugs or herbs for the treatment of cardiovascular disease (CVD) have been studied (Table 1), but the specific pharmacological activity and the mechanism(s) of action of these drugs, as well as the pathological changes that affect human diseases, need to be further investigated. A better understanding of the cellular interactions of unfolded protein response (UPR)/ER stress and other specific pathways in different vascular beds contributing to pathogenic conditions will contribute to developing new therapies to overcome CVD.

## Figures and Tables

**Table 1:** Drugs or herbs affecting endoplasmic reticulum stress to ameliorate cardiovascular diseases.

Drugs or Herbs	Action Mechanism	Cardiovascular Disease	References
4-phenyl butyric acid (PBA)	↑ insulin sensitivity; ↓ blood pressure, fatty liver disease, cardiomyocyte UPR activity, cardiac fibrosis, TGF-β1, cPLA2/COX, arterial remodeling, right ventricular hypertrophy.	Diabetes and obesity, hypertension, pulmonary arterial hypertension	[61,118,119,128,129,130]
taurine-conjugated ursodeoxycholic acid (TUDCA)	↑ insulin sensitivity, endothelium-dependent relaxation; ↓ blood pressure, fatty liver disease, cardiomyocyte UPR activity, cardiac fibrosis, TGF-β1, cPLA2/COX, Nox2, Nox4, body weight.	Diabetic vasculopathy, diabetic retinopathy, hypertension	[61,118,119,128,129,130]
Metformin	↑ AMPK, PPARδ, eNOS activity, NO production; ↓ ER stress, oxidative stress.	Diabetic vasculopathy	[91,92,93]
AG1478	↑ endothelium-dependent relaxation; ↓ Nox2, Nox4.	Diabetic retinopathy	[101]
Mangiferin	↑ TXNIP, NLRP3 inflammasome, IL-1β, IL-6, NO.	Diabetic vasculopathy	[103]
nicorandil	↑ insulin resistance; ↓ PERK inhibition.	Diabetes	[62]
Kaempferol	↑ caspase-3, caspase-7; ↓ GRP78, CHOP.	Atherosclerosis	[112]
Atrovastatin	↑ AMPK.	Atherosclerosis	[113]
hydrogen sulfide	chemical chaperones.	Pulmonary arterial hypertension	[115]
intermedin	chemical chaperones.	Pulmonary arterial hypertension	[131]
4u8c	chemical chaperones;↓ IRE1α/XBP1 inhibition.	Pulmonary arterial hypertension	[132]
black tea	↓ ER stress, oxidative stress, blood pressure, endothelial dysfunction.	Hyperhomocysteinemia	[120]
Enalapril	↓ ER stress, blood pressure.	Hyperhomocysteinemia	[148]
Piceatannol	↓ apoptosis, oxidative stress, ER stress.	Hyperhomocysteinemia	[149]
Atorvastatin	↑ AMPK; ↓ ER stress.	Hyperhomocysteinemia	[150,151]
salidroside	↓ Bip, CHOP, PERK phosphorylation, IRE1α phosphorylation.	Hyperhomocysteinemia	[152]
Alpha-lipoic acid	↓ ER stress, oxidative stress, apoptosis, inflammation.	Hyperhomocysteinemia	[153]
Exendin-4	↑ AMPK; ↓ ER stress, superoxide anion production.	Hyperhomocysteinemia	[154].
Zinc finger protein ZBTB20	↓ TNFα, ASK1, JNK, NADPH oxidase.	Myocardial infarction	[161]
Valsartan	↓ ASK1.	Myocardial infarction	[162]
*Panax notoginseng* flower	↑ HIF-1, VEGFA, KDR, Bcl-2, Bax.	Myocardial infarction	[163]
Taurine	↓ TF-6 and IRE-1.	Stroke	[169]
Apelin-36	↓ CHOP/GRP78.	Stroke	[170]
Basic fibroblast growth factor	↑ PI3K/Akt, ERK1/2; ↓ CHOP, ATF6.	Stroke	[171]
